# Co-culture of primary human tumor hepatocytes from patients with hepatocellular carcinoma with autologous peripheral blood mononuclear cells: study of their in vitro immunological interactions

**DOI:** 10.1186/1471-230X-13-17

**Published:** 2013-01-18

**Authors:** Polyxeni P Doumba, Marilena Nikolopoulou, Ilias P Gomatos, Manousos M Konstadoulakis, John Koskinas

**Affiliations:** 1 2nd Department of Internal Medicine, Medical School of Athens, University of Athens, Hippokration Hospital, 114 Vas. Sofias Avenue, Athens, 171 23, Greece; 2 1st Department of Propaedeutic Surgery, Laboratory of Surgical Research, Medical School of Athens, Hippokration Hospital, Athens, Greece

**Keywords:** HCC/non-HCC-Hepatocyte-PBMCs co-cultures, CD8+ T lymphocyte activation, Apoptosis, MHC-II expression

## Abstract

**Background:**

Many studies have suggested that the immune response may play a crucial role in the progression of hepatocellular carcinoma (HCC). Therefore, our aim was to establish a (i) functional culture of primary human tumor hepatocytes and non-tumor from patients with hepatocellular carcinoma (HCC) and (ii) a co-culture system of HCC and non-HCC hepatocytes with autologous peripheral blood mononuclear cells (PBMCs) in order to study in vitro cell-to-cell interactions.

**Methods:**

Tumor (HCC) and non-tumor (non-HCC) hepatocytes were isolated from the liver resection specimens of 11 patients operated for HCC, while PBMCs were retrieved immediately prior to surgery. Four biopsies were obtained from patients with no liver disease who had surgery for non malignant tumor (normal hepatocytes). Hepatocytes were either cultured alone (monoculture) or co-cultured with PBMCs. Flow cytometry measurements for MHC class II expression, apoptosis, necrosis and viability (7AAD) were performed 24 h, 48 h and 72 h in co-culture and monocultures.

**Results:**

HCC and non-HCC hepatocytes exhibited increased MHC-II expression at 48h and 72h in co-culture with PBMCs as compared to monoculture, with MHC II-expressing HCC hepatocytes showing increased viability at 72 h. PBMCs showed increased MHC-II expression (activation) in co-culture with HCC as compared to non-HCC hepatocytes at all time points. Moreover, CD8+ T cells had significantly increased apoptosis and necrosis at 48h in co-culture with HCC hepatocytes as compared to monocultures*.* Interestingly, MHC-II expression on both HCC and non-HCC hepatocytes in co-culture was positively correlated with the respective activated CD8+ T cells.

**Conclusions:**

We have established an in vitro co-culture model to study interactions between autologous PBMCs and primary HCC and non-HCC hepatocytes. This direct interaction leads to increased antigen presenting ability of HCC hepatocytes, activation of PBMCs with a concomitant apoptosis of activated CD8+ T cells. Although, a partially effective immune response against HCC exists, still tumor hepatocytes manage to escape.

## Background

The effective immune response against hepatocellular carcinoma is of great importance in the progression and clearance of the tumor. Many studies have shown that the immune response against HCC is restrained and not effective [1-3]. Cytotoxic T lymphocytes (CTLs) play a central role in induction of anti-tumor immunity. Indeed, a high frequency of cytotoxic CD8+ T cells infiltrating cancer tissue can be a favorable prognostic indicator in HCC [4]. However, the progression of tumors despite the presence of infiltrating or peripheral cytotoxic CD8+ T cells suggests that immunological tolerance is induced, at least in part, by tumors.

Tumor-specific immune responses were observed in a significant proportion of patients with HCC. However, spontaneous clearance of established tumors by endogenous immune mechanisms is rare [5]. The fact that many tumor-associated antigens (TAA) are normal self-constituents, the immune responses that may generate are generally weak. Moreover, the pressure of the host immune system may lead to the development of various mechanisms by which the tumor cells escape from immune attack [6].

The induction of MHC-II expression on hepatocytes would it be a possible mechanism of inducing the defective immune response against many liver diseases including HCC. Several studies have reported that hepatocytes express MHC II molecules and are able to activate CD4+ and CD8+ T cells [7-9].

Although, in vitro and in vivo studies have demonstrated that hepatoma cells may express high levels of both class I and class II molecules [10-12], little is known about their potential immunogenicity [13-15]. Currently, there is no evidence on the immunologic outcome of the direct interaction between malignant human hepatocytes and circulating T lymphocytes in an in vitro co-culture system. The existing liver sinusoid barrier, as well as the complex liver microcirculation hinders examination of the direct interaction between these two cell populations in vivo. Therefore, the establishment of a human experimental in vitro co-culture system for the study of the interaction between HCC hepatocytes and PBMCs is of great importance since all the evidence concerning the anti-tumor response to HCC is based on animal models.

Here we report the establishment of a functional co-culture system of HCC and non-HCC hepatocytes, isolated from the transected liver specimens of patients submitted to anatomic liver resection for primary HCC, with autologous PBMCs in order to study their direct effect. In addition, these two types of cells interact with each other leading to increase MHC-II expression on both HCC and non-HCC hepatocytes with a concomitant activation of CD8+ T cells, which finally become apoptotic.

## Methods

### Patients

Eleven patients, 10 males and 1 female, age between 57–75 years with cirrhosis and early stage HCC have been submitted to anatomic liver resection with curative intent; 5 patients had chronic HBV infection, 2 alcoholic liver disease, 1 NASH and in 3 patients the reason of liver damage could not be defined (cryptogenic). All patients have been operated in the Surgical Unit of Hepatopancreatobiliary Diseases of the 1st Department of Propaedeutic Surgery of the University of Athens, between December 2005 and December 2010. Consent has been retrieved from our patients, while the protocol has been approved by the Ethical and Scientific Committee of our Hospital. Liver specimens (from tumor and non-tumor regions) and preoperative peripheral blood samples were obtained from all our patients. Also, four biopsies were obtained from patients with no liver damage who had surgery for non malignant tumors (healthy, normal hepatocytes).

### Isolation and culture of PBMCs

Prior to liver tissue resection 10 to 15 ml of autologous peripheral blood was collected in EDTA tubes and PBMCs were isolated by using Histopaque 1.077 g/ml (Sigma Chemicals, Germany), as previously described [16]. The resulted cell pellet (PBMCs) was resuspended in culture medium containing DMEM-HAM F12 (Biochrom co, Germany) supplemented with 1% FBS (Gibco BRL, USA), 0.25 mg/ml human insulin (Sigma Chemicals, Germany), 100 U/ml Pen/Strep, 50 μg/ml gentamycin (Sigma Chemicals, Germany), 20 mM HEPES (Gibco BRL, USA) and 1.3 mM L-Glutamine (Biochrom co, Germany) and the yield of PBMCs was determined by Neubauer enumeration.

The above culture medium was chosen after experiments that were performed for both hepatocytes and PBMCs, in order to find the optimal co-culture conditions. Therefore, hepatocytes and PBMCs were maintained in monocultures using different FBS concentrations 0%, 1% and 5% in DMEM-F12 supplemented with 0.25 mg/ml human insulin, 100 U/ml Pen/Strep, 50 μg/ml gentamycin, 20 mM HEPES and 1.3 mM L-Glutamine for 7 days*.* The viability of both hepatocytes and PBMCs after 7 days in culture was high, always exceeding 75%. The optimum FBS concentration for the culture medium was the 1%. PBMCs were then plated in 60-cm culture dish until the day of the co-culture experiment in 1% culture medium.

### Isolation and culture of HCC and non-HCC hepatocytes

Tumor liver specimens were obtained from the primary tumor and non-tumor surrounding liver parenchyma and transferred in ice-cold DMEM-F12 supplemented with 10% FBS. Hepatocyte isolation was performed based on previous isolation protocol with modifications [17-19].

Liver specimens were treated with pre-warmed 0.1% Collagenase Type IV (Sigma Chemicals, Germany) and filtered through a 70 μm cell strainer (BD, USA). The pellet containing the liver cells was resuspended in ice-cold DMEM-F12 with 20%FBS and triple centrifugation at 81 g for 12 min at 4°C was performed in order to separate the purified hepatocyte population (pellet) from the non-parenchymal cells (supernatant) [20].

The pellet contained the purified hepatocytes, as further confirmed by light microscopy and by flow cytometric analysis of the two hepatocyte specific markers Hep-Par 1 and albumin described in the next paragraphs. Next, hepatocytes remained in culture for few hours in 60-mm dishes without collagen I in order to achieve maximum hepatocyte purity, by removing possible remaining non-parenchymal cells. Then, we carefully removed the supernatant containing the hepatocytes and 1 × 10^5^ tumor and non-tumor hepatocytes were placed in 24-well plates coated with 5 μg/cm^2^ rat tail collagen type I (BD, USA) in 10% FBS culture medium and allowed to adhere for 48 h.

16 hours prior to co-culture experiment the hepatocyte medium was removed and hepatocytes were washed twice with 1 × PBS in order to remove the possible remaining intrahepatic lymphocytes. Then, 1% FBS culture medium was added to the hepatocytes. The same procedure was performed for the normal liver biopsies.

### Co-cultures of HCC and Non-HCC Hepatocytes with PBMCs

After initial plating the culture medium was carefully aspirated. The ratio of PBMCs (effector cells) to hepatocytes in co-cultures was 5:1. The ratio that was chosen was based on data from the literature. Most studies that involved co-culture of rat lymphocytes with autologous hepatocytes used the ratio of 10:1 [21-23]. We thought that a ratio of 5:1 would be more realistic to seek interactions between HCC and non-HCC hepatocytes with autologous peripheral immune cells.

PBMCs were collected and washed by centrifugation. The cell pellet was resuspended in 1% FBS medium and the appropriate number of mononuclear cells was added in each well on top of the adhered hepatocytes.

PBMCs with HCC-hepatocytes as well as PBMCs with non-HCC hepatocytes were cultured for 24 h, 48 h and 72 h. As control cultures tumor, non-tumor hepatocytes and PBMCs were maintained in monocultures. The same procedure was performed for the four normal hepatocyte PBMCs co-cultures.

### Expression of Hep Par-1 on hepatocytes by flow cytometry

After isolation, normal, HCC and non-HCC hepatocytes were stained for the hepatocyte specific marker Hep Par-1 in order to verify the purity of the hepatocyte population.

Hepatocytes were fixed with 4% paraformaldehyde (PFA) (Sigma, chemicals, Germany) and centrifuged in 1 × PBS (Cambrex co, USA). Hepatocytes were then permeabilized with 0.1% Triton X-100 (Sigma, chemicals, Germany). Hep Par-1 antibody (Dako Cytomation DK) (1:10 dilution) was incubated with Zenon ALEXA Fluor-488 (Molecular Probes, UK) prior to addition to the cell suspension. Then, hepatocytes suspension was incubated with the Hep Par-1/Zenon ALEXA Fluor-488 antibody mixture. The cells were then immediately analyzed by using EPICS-XL MCL flow cytometer. Unstained cells were used for the determination of the positive stained hepatocyte population.

### Hep Par-1 immunocytochemistry

Cytospin preparations of hepatocyte’s suspension were fixed with 4% PFA. Hepatocytes were washed with 6% BSA (Sigma, chemicals, Germany) /0.2% Tween-20 (Sigma, chemicals, Germany) and 0.1% Triton X-100 was used for the permeabilization step. After a washing step, blocking was performed with hydrogen peroxidase (1:10 dilution). Hepatocytes were incubated with Hep Par-1 antibody (1:5 dilution). The Envision Peroxidase Detection Kit (Dako Cytomation, DK) was used in order to detect Hep-Par-1. Hematoxylin was used for staining the nuclei. Hepatocytes were then observed using microscope Zeiss Axiostar plus.

### Analysis of MHC class II expression and viability of tumour and non-tumour hepatocytes by flow cytometry

The supernatant from HCC non-HCC and normal co-cultures, containing the PBMCs population was carefully removed. The adhered hepatocytes were detached from the wells by trypsinization at 24 h, 48 h and 72 h after co-culture. In addition, hepatocytes were treated with Stem Pro Accutase (Life technologies, Gibco, USA) in order to avoid the formation of cell clusters.

The same procedure was performed for the monocultures as well.

The hepatocyte pellets were triple stained with the HLA-DR, DP, DQ-PE (Beckman Coulter, USA) (MHC class II-PE) (surface), 7-AAD (BD, USA), as well as with anti-human albumin-FITC (Dako Cytomation, DK) (intracellular).

After isolation, hepatocytes were also stained with anti-CD3 (Beckman Coulter, USA) and anti-CD14 (Beckman Coulter, USA) monoclonal antibodies to further verify their purity. MHC class II expression and 7-AAD measurements were also performed after isolation. Hepatocytes were immediately analyzed by flow cytometry. Unstained hepatocytes were used as controls in order to determine the autofluorescence of the cells and for setting the flow cytometry protocol.

### Flow Cytometric analysis of PBMCs

CD8+ T cells were characterized from the total PBMCs by staining them with the surface marker CD8-FITC (Beckman Coulter, USA). The activation of both mononuclear cells and CD8+ T cells was estimated by the surface *activation* marker HLA-DR, DP, DQ [24]. Viability, apoptosis and necrosis of PBMCs and CD8+ T lymphocytes was determined by 7-AAD staining. The above measurements were performed by flow cytometry after isolation, before co-culture experiment, 24 h, 48 h and 72 h post co-culture. The same measurements took place for the PBMCs monocultures as well.

As mentioned before the supernatants from tumor and non-tumor co-cultures contained the PBMCs population. Mononuclear cells were carefully aspirated from each well and centrifuged. Cells were then triple stained with the monoclonal CD8-FITC antibody, HLADR, DP, DQ-PE and 7-AAD. Unstained PBMCs were used as controls in order to determine the autofluorescence of the cells.

### Statistical analysis

All continuous variables are reported as the mean (± SD). Paired comparisons between qualitative variables were performed using the Wilcoxon sign rank test. Data were analyzed using SPSS 13.0 software*.* Pearsons correlation coefficient was used to detect any positive or negative correlations. P values <0.05 were considered statistically significant.

## Results

### Viability and albumin expression of hepatocytes

The cell yield per gram of wet tissue after Collagenase IV treatment varied between the different tissue samples due to different underlying disease condition and age. In average the cell yield was 7 × 10^6^ for the HCC-hepatocytes and 5 × 10^5^ for the non-HCC hepatocytes. Immediately post-isolation the average viability was 90% and 92% for HCC and non-HCC hepatocytes, respectively. HCC and non-HCC hepatocytes expressed albumin protein at the level of 94% and 92%, respectively as documented by means of flow cytometry, which remain at the same levels throughout the experiment. Healthy hepatocytes exhibited increased cell yield, viability and albumin levels.

**Figure 1:**
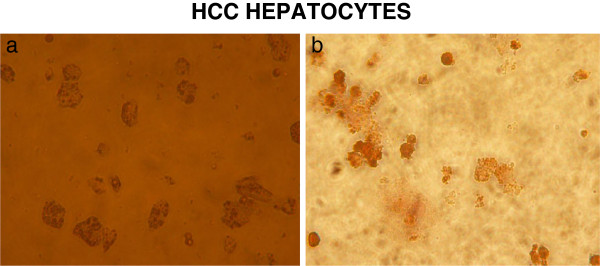
a) Representative photos of HCC hepatocytes in culture 48 hours after isolation (magnification x 20) and b) HCC hepatocytes stained with Hep Par-1 after isolation (immunocytochemistry) (magnification × 20).

**Figure 2:**
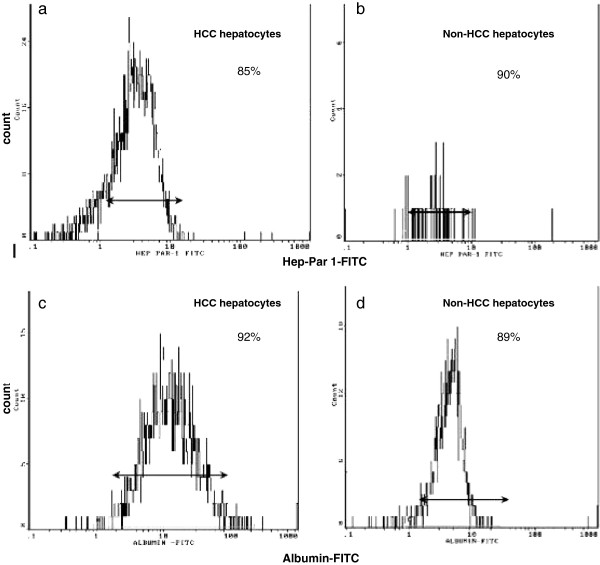
Flow cytometry diagrams presenting: a) percentage of HCC-tumor hepatocytes and b) percentage of non-HCC hepatocytes stained positive for Hep Par-1, using the secondary antibody Zenon-488, after isolation c) percentage of HCC-tumor and d) percentage of non-HCC hepatocytes stained for human albumin-FITC.

**Figure 3:**
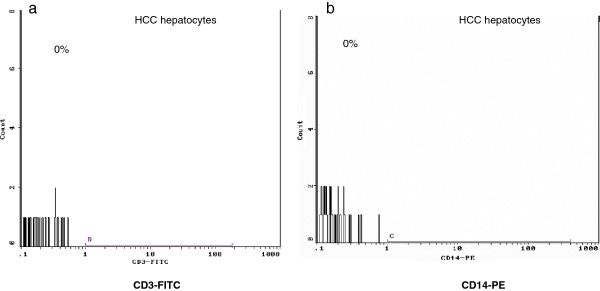
After isolation tumor, non-tumor and normal hepatocytes were stained for CD3 and CD14 markers and analyzed by flow cytometry in order to verify the purity of the hepatocyte culture was performed; representative flow cytometric diagram of a) CD3-FITC and b) CD14-PE negative staining in tumor hepatocytes.

The morphological characteristics of HCC and non-HCC hepatocytes remained grossly intact in culture conditions, with viable hepatocytes presenting typical and undamaged morphology as demonstrated by light microscopy (Figure 1a). Freshly isolated hepatocytes attached to collagen-coated dishes after initial plating and formed semi-confluent monolayers within 48 h. Hep Par-1 immunocytochemistry (Figure 1b), and flow cytometry panels (Figure 2a,b) demonstrated hepatocellular purity at the level of 85% to 90% for both HCC- and non-HCC-cell cultures. The functionality of HCC and non-HCC hepatocytes was measured by anti-human albumin-FITC and was 92% and 89%, respectively (Figure 2c,d). Negative staining for CD14, CD3 further confirmed the absence of Kupffer cells and mononuclears cells in the co-culture system (Figure 3a,b).

### MHC-II expression and viability of HCC and non-HCC hepatocytes in co-culture with autologous PBMCs

After isolation, the MHC II expression on HCC hepatocytes was higher compared to non–HCC hepatocytes, although this did not reach statistical significance (20% vs 15%). On the other hand, MHC II expression on hepatocytes derived from individuals with no liver disease had significant lower MHC II expression, ranged between 3%-8%.

In co-culture with autologous PBMCs, the expression of MHC class II molecules on hepatocytes was further increased compared to the single cultures at all time points both for HCC and non-HCC hepatocytes.

**Figure 4:**
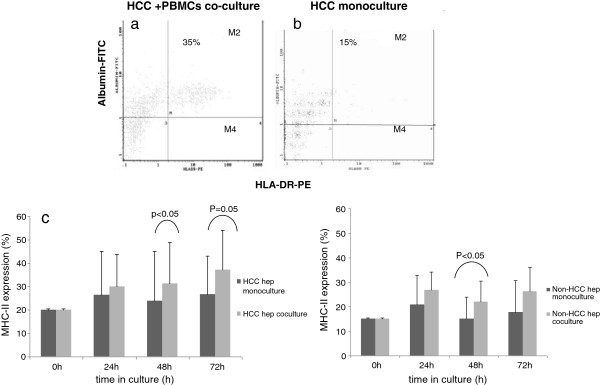
**Representative flow cytometry scatterplot depicting the increased expression of MHC II on HCC hepatocytes (M region) a) 48 hours in co-culture with autologous PBMCs vs. b) 48 hours in monoculture.** Representative graph showing the percentage of MHC II expression on **c**) HCC and **d**) non-HCC hepatocytes in co-culture compared to hepatocyte monocultures. Time point zero (0 h) depicts MHC II expression on hepatocytes directly after isolation.

For the HCC hepatocytes the respective difference reached statistical significance at 48 h in co-culture compared to monocultures (31.4 vs 23.8, p < 0.05), with a further increase at 72 h (37.1 vs 28.1, p = 0.05) (Figure 4a, b, c).

Non-HCC hepatocytes also exhibited significant increased MHC II expression 48 h post-co-culture with PBMCs in comparison to mono-culture (21.9 vs 15, p < 0.05) (Figure 4d).

Moreover, the expression of MHC class II molecules on HCC hepatocytes was higher at all time points compared to non-HCC hepatocytes both in co-cultures and monocultures.

MHC II expression on hepatocytes from individuals with no liver disease remained the same for both monocultures and co-cultures with autologous PBMCs at all time points (3-5%).

Interestingly, the viability of total non-HCC hepatocytes in co-culture was significantly lower at all time points compared to the respective hepatocytes in monocultures (24 h: 27.1 vs 63.8, p < 0.05, 48 h: 10.4 vs 62.6, p < 0.05 and 72 h: 27.4 vs 55.8, p < 0.05). In contrast, the viability of total HCC hepatocytes had not difference compared to the respective hepatocytes in monocultures.

The viability of MHC II-expressing HCC hepatocytes after 72 h in co-culture with PBMCs was significantly higher compared to MHC II-expressing HCC hepatocytes in monoculture (11.5 vs 8.2 p < 0.05). In contrast, MHC II-expressing non-tumor hepatocytes showed a non-significant increased viability in co-culture compared to their monocultures.

### Effects of HCC and non-HCC hepatocytes on PBMCs and CD8+ T cells

**Figure 5:**
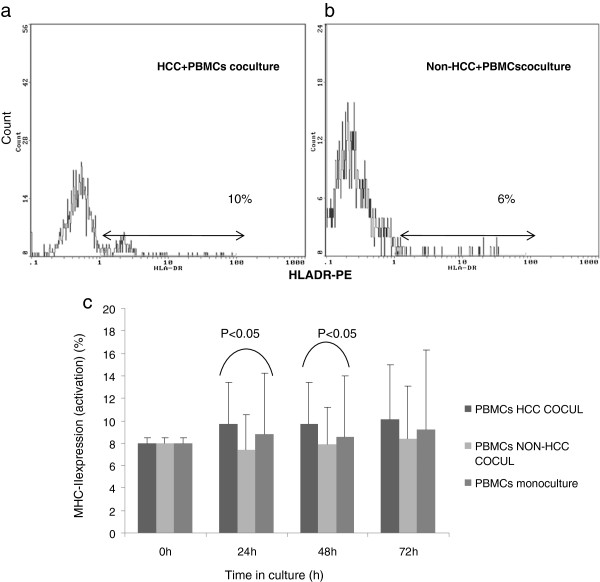
**Flow cytometric diagrams showing total PBMCs activation (MHC-II expression) after 48 h in co-culture with a) HCC and b) non-HCC hepatocytes. c**) PBMCs exhibited significant activation at 24 h and 48 h when co-cultured with HCC hepatocytes compared to non-HCC hepatocytes. Also, the activation of PBMCs in HCC co-culture was higher compared to PBMCs monoculture. Time point zero (0 h) depicts activation of total PBMCs after isolation (0 h).

The median expression of MHC II (activation) on PBMCs from patients with HCC after isolation was 8%. PBMCs exhibited an increase of MHC II expression in co-culture with HCC as compared to non-HCC hepatocytes at all time points. This difference in mononuclear cells activation was found to be statistically significant at 24 h (9.7 vs 7.4, p < 0.05) and 48 h (9.7 vs 7.9 p < 0.05) (Figure 5a, b, c).

Although, MHC II expression on PBMCs was elevated in co-culture with tumor hepatocytes compared to PBMCs monocultures, the respective differences did not reach statistical significance (Figure 5c). In contrast, in non-HCC co-culture there was no evidence of increased MHC II expression on PBMCs at all time points, at least with the activation markers that we have used.

Also, no differences were observed concerning the apoptosis and necrosis of activated and total peripheral mononuclear cells in both HCC and non-HCC hepatocyte co-cultures compared with PBMCs monocultures.

Further, we examined the behavior of CD8+ T cell subset from the total PBMCs population when co-cultured with HCC and non-HCC hepatocytes. Therefore, in the total PBMCs population we performed double staining for CD8+ and MHC II expression (CD8 + MHC II+, phenotype of activated CD8 + T cells).

**Figure 6:**
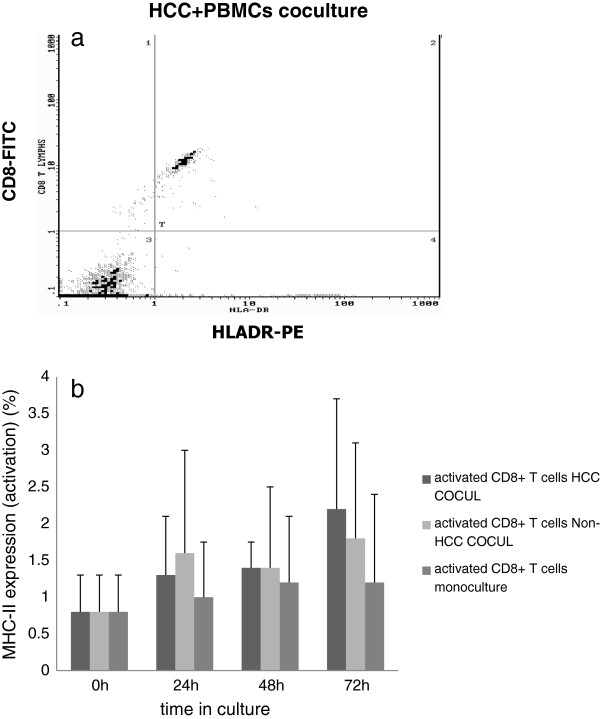
**a) Representative flow cytometric diagram showing the increased expression of MHC class II on CD8+ T cells (activated) at 72 h HCC co-culture, b) CD8+ T cells exhibited higher activation at all time points compared to control PBMCs culture with a peak at 72 h.** Also, the activation of CD8+ T cells in both HCC and non-HCC cocultures was higher compared to CD8+ T cells activation after isolation (0h).

CD8+ T cells showed an increase of MHC II expression at all time points with a maximum at 72 h in co-culture with HCC hepatocytes compared to PBMCs monoculture although statistically was not significant (Figure 6a, b). No significant activation of CD8+ T cells was observed in co-culture with non-HCC hepatocytes (Figure 6b).

**Figure 7:**
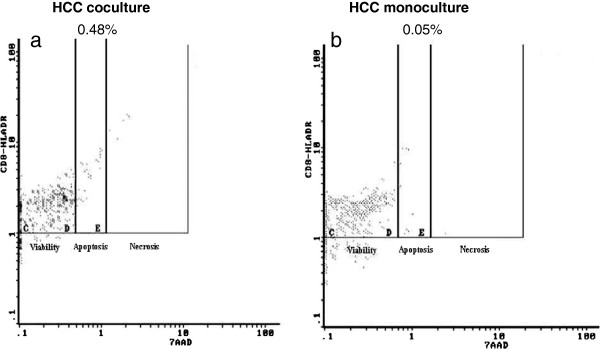
Flow cytometric diagrams showing the % of activated CD8+ T cells (CD8+ MHC II) that are apoptotic (D region) and necrotic (E region) in a) HCC co-cultures and b) in control PBMCs cultures.

7-AAD analysis of CD8+ T cells co-cultured with HCC or non-HCC hepatocytes revealed an increased necrosis at 48 h (p < 0.05) as compared to control (PBMCs monocultures). The activated CD8+ T cells (MHC II+) also showed increased apoptosis and necrosis in co-culture with HCC hepatocytes compared to controls especially at 48 h (0.18 vs 0.06, p = 0.046 and 0.15 vs 0.05, p < 0.05) (Figure 7). This phenomenon was not observed in non-HCC co-cultures.

In the co-cultures of hepatocytes from individuals with no liver disease with their autologous PBMCs no changes were observed in activation or in viability status of either PBMCs or CD8+ T cells.

We next examined the correlation between the MHC-II expression on HCC and non-HCC hepatocytes and activation of CD8+ T cells and PBMCs. Indeed, we found that the MHC-II expressing HCC hepatocytes were positively correlated with the activated CD8+ T cells (CD8 + MHC-II+) after 24 h and 48 h in HCC co-culture (r = 0.947, p < 0.001 and r = 0.878, p = 0.021, respectively). In addition, MHC-II expressing non-HCC hepatocytes were also positively correlated with the activated CD8+ T cells after 24 h and 72 h in co-culture (r = 0.0.925, p = 0.008 and r = 0.948, p = 0.014, respectively).

## Discussion

We have established a functional culture system for HCC and non-HCC hepatocytes originating from surgical tissue specimens retrieved from HCC patients undergoing hepatectomy with intention to cure. 7-AAD and human albumin staining indicated high viability and functionality of hepatocytes (HCC, non-HCC and healthy) in vitro, respectively. Hepatocyte purity after isolation with the isolation protocol that we used was exceeding 95% and was demonstrated by positive Hep Par-1 and negative CD3 and CD14 immunostaining.

Data from the literature indicate that the outcome of interactions between tumor hepatocytes and immune cells is critical for cancer regression or progression. There is lack of appropriate models to address how human immune system interacts with liver cancer cells. With the restriction of human experimental system, most knowledge on liver cancer immunity is derived from animal models. So, we have established an in vitro co-culture system of tumor and non-tumor hepatocytes with PBMCs isolated from peripheral blood drawn prior to resection in order to study their immunological interactions. With restrictions we believe that this in vitro system will provide to us information of what happens in vivo.

There are many scientific reports which have shown that hepatocytes express MHC class II molecules and can act as antigen presenting cells. Although, hepatocytes can activate CD4+ and CD8+ T cells there are not able to sustain this activation and to create an effective immune response [7-9]. In addition, MHC molecules may be either expressed de novo or up-regulated on a number of tumor cells [25,26], but any relation to an effective presentation of tumor antigen to T cells for stimulation and immunological rejection of tumors is controversial. Although many tumors express rejection antigens as well as MHC molecules they avoid destruction by T cells [27-33].

Therefore, it is of great importance to study if primary human neoplastic hepatocytes derived from HCC tumors are able to express MHC class II molecules and have the ability to act as antigen presenting cells when they are in contact with peripheral blood mononuclear cells. If this is true, HCC hepatocytes will be able to provide help to immune cells, which are already defective, both in the periphery and in the tumor microenvironment*.*

Under normal conditions, hepatocytes do not express MHC II molecules. However, hepatocellular MHC class II expression has been reported in patients with hepatitis [34,35], as well as in patients with HCC [10,11]. Furthermore, tumor hepatocytes from hepatoma cell lines express MHC class II molecules and could act as antigen presenting cells [15,35]. Recent experimental evidence suggests that the liver acts as a site of primary T-cell activation leading to functional impairment and premature death of cytotoxic T cells [36]. In an experimental animal study by Bertolino *et al*. [7], hepatocytes were capable of inducing in vitro activation and proliferation of naive CD8+ T cells, in the absence of CD4+ T cell co-stimulation, a clear indication that they possess the ability of antigen presentation.

Indeed, according to our results, HCC hepatocytes exhibited high expression of MHC II on their surface compared to non-HCC hepatocytes, expression that is further enhanced upon co-culture with autologous PBMCs at all time points. Increased MHC-II expression was also observed in the non-HCC hepatocytes in co-culture with PBMCs. However it has to be addressed that the non–HCC hepatocytes are not really healthy as they have derived from the HCC patients with underlying viral or other etiology chronic liver disease. In contrast, hepatocytes derived from individuals with no liver disease had minimal MHC class II expression at the beginning, which remained at the same low levels at all time points in the monoculture or co-culture with PBMCs system.

With the respect to the effect of hepatocytes on PBMCs our experiments showed that the expression of MHC II on total PBMCs, which indicates an activation status, was statistically higher in the presence of HCC than non-HCC cells, particularly after 24 h and 48 h of incubation. Furthermore, CD8+ T cell activation was clearly seen after 72 h in the HCC co-culture system than in the non-HCC system. No effect on MHC II expression on PBMCs and CD8+ T cells was observed in the non-HCC co-culture system.

We next examine if the MHC-II expression on the tumor and non-tumor hepatocytes in co-culture had any correlation with the activation status of the CD8+ T cells or the total PBMCs. Interestingly, we found that the expression of MHC-II on HCC hepatocytes was positively correlated with the activated CD8+ T cells 24 h and 48 h post-coculture. The same correlation was also true for the non-HCC hepatocytes but at different time points (24 h and 72 h post-coculture).Therefore, both MHC-II expression on HCC and non-HCC hepatocytes in co-culture was directly linked to the activated CD8+ T cells, leading to effective immune response.

In agreement to these results is the fact that γ-interferon (IFN-gamma) produced by T lymphocytes that infiltrate the liver during the course of chronic hepatitis induces MHC II expression and may endow the hepatocytes with the capacity to perform accessory, antigen-presenting, cell functions [35].

There is a possibility that a similar effect could also happen in our in vitro co-culture system. In this respect IFN-γ could possibly have a dual effect on HCC hepatocytes in co-culture; destruction of tumor cells and over-expression of MHC II on them. This may also true for the non-HCC virus infected hepatocytes. However, the viability of MHC II-expressing HCC cells was significantly higher in the co-culture system as compared to the monocultures at 72 h, observation that was not evident in non-HCC cells. In addition to the above observation, the total viability of HCC hepatocytes in co-culture remains unchanged, while the viability of the respective non-HCC hepatocytes was significantly lower at all time points compared to the respective monoculture. Therefore, this further enhanced the idea that HCC hepatocytes managed to survive and escape from the immune attack in contrast to the respective non-HCC that exhibited lower viability.

As a possible examination is the fact that increased necrosis at 48 h in the total CD8+ T lymphocytes in the HCC co-culture compared to PBMCs monoculture was seen. More importantly, at 48 h, the activated CD8+ T cells had undergone significant apoptosis and necrosis compared to controls. However, the factors and the mechanisms involved could be beyond the elimination of the activated CD8+ T cells.

Indeed, several studies have shown that tumor cells expressing decoy molecules, such as DcR3, can escape FasL-dependent immune-cytotoxic attack. Thus, DcR3 is considered to be one of the immune evasion systems for tumor progression [37]. Also, it has been reported that the number of regulatory T cells in the peripheral blood and in the tumor microenvironment is increased and that may exerts an inhibitory effect in the function of the immune cells, including CD8+ T cells, [38], offering an additional escape mechanism to HCC hepatocytes.

The elimination of CD8+ T cells here is not due to death by neglect or lack of secondary activation signals (cytokines etc.), as it was referred in other studies [7]. In our co-culture system, other immune cells are present which could provide CD8+ T cells with secondary signals and support CD8+ T cell activation something that is verified from the increased activation of PBMCs population.

## Conclusions

In conclusion we have established, for the first time, an in vitro model to study the interaction between autologous PBMCs and CD8+ T cells with primary HCC and non-HCC hepatocytes.

Our results revealed a very interesting counteraction; an increased expression of MHC II on both HCC and non-HCC hepatocytes and activation of PBMCs followed by an enhanced viability of MHC-II-expressing HCC cells with a concomitant apoptosis of the activated CD8+ T cells. Further experiments should take place in order to understand the mechanisms of this interaction and benefit from this ability of tumor hepatocytes to act as antigen presenting cells and to activate the immune cells.

## Abbreviations

PBS: Phosphate buffer saline; 7-AAD: 7-actinomycin-D; FBS: Fetal bovine serum; HCC: Hepatocellular carcinoma; NASH: Non- alcoholic steatohepatitis; Pen/Strep: Penicillin/streptomycin.

## Competing interests

The authors declare that they have no competing interests.

## Authors’ contribution

JK (Associate Professor of Internal Medicine-Hepatology), has designed the protocol, supervising in all the steps of the study and contributed to the final form of the manuscript. MMK (Associate Professor of Surgery), has contributed to the collection of the surgical human liver specimens, the design of the protocol and supervising in all the steps of the study. PPD, (Biochemist, PhD student), has done the research work and has written the paper. MN, (Biologist, PhD), has contributed in the lab work. IPG, (MD, PhD), has contributed partially in the collection of surgical liver specimens and has done the statistical analysis. All authors have contributed significantly and agree with the content of the manuscript.

## Pre-publication history

The pre-publication history for this paper can be accessed here:

http://www.biomedcentral.com/1471-230X/13/17/prepub
